# CPX-351 in *FLT3*-mutated acute myeloid leukemia

**DOI:** 10.3389/fonc.2023.1271722

**Published:** 2023-11-17

**Authors:** Claire Andrews, Vinod Pullarkat, Christian Recher

**Affiliations:** ^1^Department of Haematology, St Vincent’s University Hospital, Dublin, Ireland; ^2^Department of Hematology & Hematopoietic Cell Transplantation, City of Hope Comprehensive Cancer Center, Duarte, CA, United States; ^3^Service d’Hématologie, Centre Hospitalier Universitaire de Toulouse, Institut Universitaire du Cancer de Toulouse Oncopole, Université Toulouse III Paul Sabatier, Toulouse, France

**Keywords:** acute myeloid leukemia, chemotherapy, CPX-351, FLT3 inhibitors, *FLT3* mutations

## Abstract

CPX-351, a dual-drug liposomal encapsulation of daunorubicin and cytarabine in a 1:5 molar ratio, is approved for the treatment of newly diagnosed therapy-related acute myeloid leukemia (AML) or AML with myelodysplasia-related changes. In a pivotal phase III trial, CPX-351 significantly improved overall survival compared with standard-of-care 7 + 3 chemotherapy (7 days cytarabine; 3 days daunorubicin) in adults aged 60–75 years with newly diagnosed high-risk or secondary AML (median = 9.56 months vs. 5.95 months; hazard ratio = 0.69; 95% confidence interval = 0.52–0.90; *p* = 0.003). Approximately 30% of patients with newly diagnosed AML have mutations in the *FLT3* gene, which may be associated with poor outcomes. Here, we review the current *in vitro*, clinical, and real-world evidence on the use of CPX-351 in patients with AML and mutations in *FLT3*. Additionally, we review preliminary data from clinical trials and patient case reports that suggest the combination of CPX-351 with FLT3 inhibitors may represent another treatment option for patients with *FLT3* mutation-positive AML.

## Introduction

CPX-351 is a dual-drug liposomal encapsulation of daunorubicin and cytarabine in a synergistic 1:5 molar ratio (1). Phase III trial data have shown CPX-351 to be significantly more effective than standard-of-care 7 + 3 chemotherapy (7 days cytarabine; 3 days daunorubicin) in treating newly diagnosed adults aged 60–75 years with high-risk/secondary acute myeloid leukemia (AML). Among 309 randomized patients, overall survival (OS) was significantly improved with CPX-351 vs. 7 + 3 (median = 9.56 months vs. 5.95 months; hazard ratio (HR) = 0.69; 95% confidence interval (CI) = 0.52–0.90; one-sided *p* = 0.003) (2). At 5-year follow-up, the median OS was 9.33 months with CPX-351 and 5.95 months with 7 + 3 (HR = 0.70; 95% CI = 0.55–0.91), and the 5-year OS rate was 18% with CPX-351 and 8% with 7 + 3 (3). CPX-351 treatment also resulted in a significantly higher overall remission rate compared with 7 + 3 (47.7% vs. 33.3%; two-sided *p* = 0.016) (2). Among patients who subsequently underwent hematopoietic cell transplantation, 3-year OS landmarked from the date of hematopoietic cell transplantation was 56% with CPX-351 and 23% with 7 + 3 (3). Based on these data, CPX-351 was approved for the treatment of newly diagnosed, therapy-related AML (t-AML) or AML with myelodysplasia-related changes (AML-MRC) in adult and pediatric patients aged 1 year and older in the USA and in adults in the European Union (4, 5). It should be noted that in the 2022 update of the World Health Organization classification for hematolymphoid tumors, the classification AML-MRC was replaced with “AML, myelodysplasia-related” (AML-MR), which requires the presence of cytogenetic or molecular abnormalities and/or a history of myelodysplastic neoplasms (MDS) or MDS/myeloproliferative neoplasms for diagnosis (6).

*FLT3* is expressed in most AML blasts and plays a key role in normal hematopoiesis and leukemogenesis (7, 8). Approximately 30% of patients with newly diagnosed AML have mutations in the *FLT3* gene, most commonly an internal tandem duplication (ITD; ~25% of cases) or point mutations in the tyrosine kinase domain (TKD; 7%–10% of cases) (9). Among patients with t-AML and normal karyotype, the frequencies of *FLT3*-ITD and *FLT3*-TKD mutations are 23% and 9%, respectively (10). The *FLT3*-ITD mutation is associated with poor outcomes in patients with AML, whereas the prognostic impact of *FLT3*-TKD mutations is unclear (9). Here, we review the preclinical and clinical evidence on the use of CPX-351 in patients with AML and mutations in *FLT3*.

## *Ex vivo*/*in vitro* data

**Figure 1:**
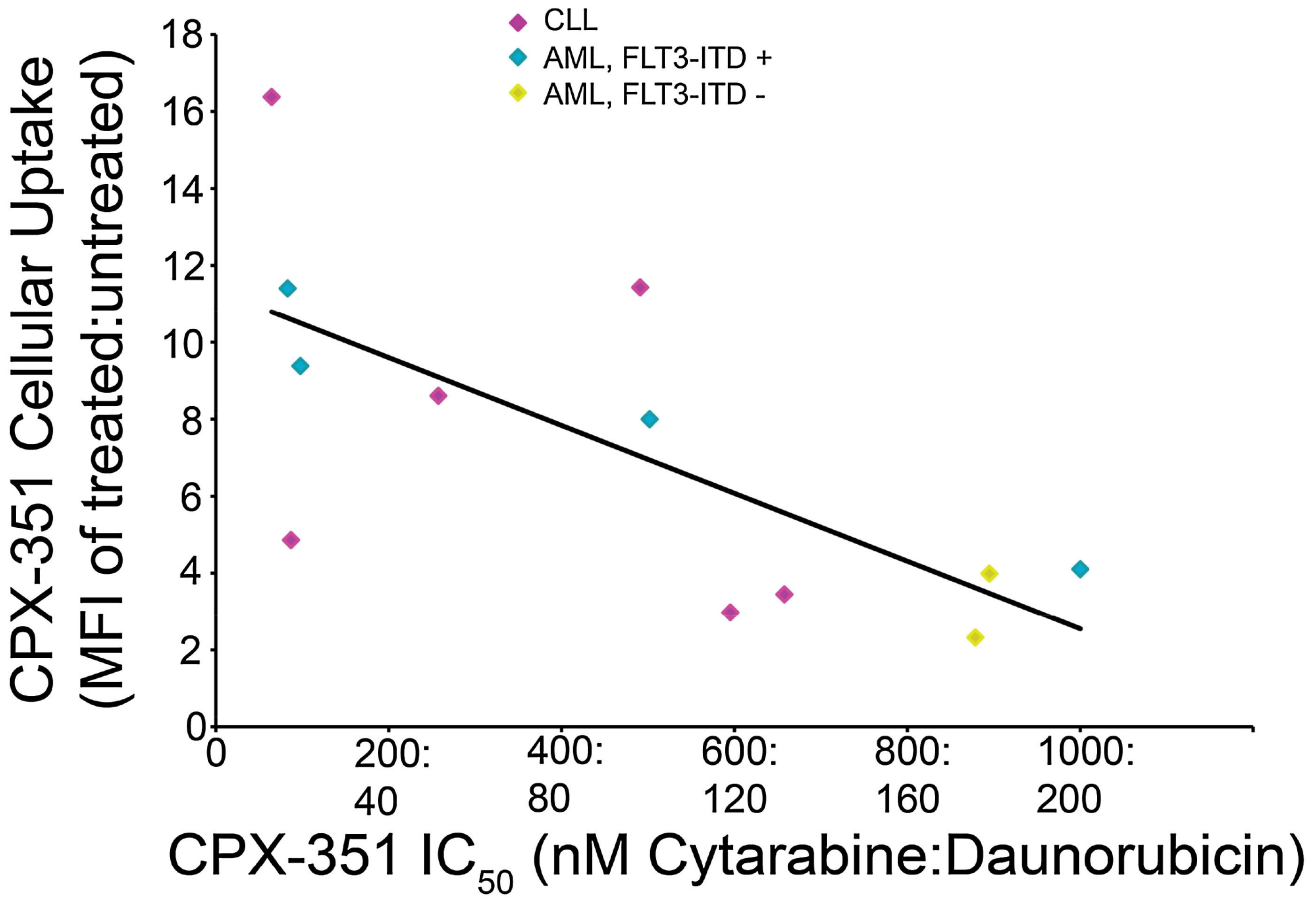
Correlation of CPX-351 cytotoxicity with cellular uptake in AML blasts. Patient blasts (*n* = 12) were exposed to graded concentrations of CPX-351 for 24 h, and the number of viable cells at 3 days was used to calculate IC_50_ values for each sample. CPX-351 uptake was assessed by analysis of daunorubicin fluorescence uptake using flow cytometry; the ratio of mean fluorescence intensity (MFI) in treated vs. untreated cells was calculated and plotted against CPX-351 IC_50_. The correlation coefficient between IC_50_ and MFI was 0.703. Figure reproduced with permission from Gordon et al. 2017 (11).

Following initial reports of CPX-351 activity compared with conventional drug combinations in phase II trials, the cytotoxic activity of CPX-351 was assessed across risk groups using an *ex vivo* assay with freshly harvested blasts from 53 patients with AML (11). Primary AML leukemia blasts showed high sensitivity to CPX-351, with 50% growth inhibition concentration (IC_50_) values ranging from 0.035:0.007 μM to 9.77:1.95 μM. Responses to CPX-351 were similar across conventional cytogenetic risk groups (per 2010 European LeukemiaNet criteria), including blasts with intermediate II or adverse cytogenetic and molecular abnormalities, and were not correlated with prior response to 7 + 3 treatment. In total, samples from 14 patients with AML were *FLT3*-ITD-positive, and these blasts were found to be nearly five times more sensitive to CPX-351-induced cytotoxicity than *FLT3*-ITD-negative AML blasts (mean IC_50_ values = 0.29:0.058 μM and 1.32:0.26 μM, respectively; *p* = 0.047 for difference). In contrast, mutations in *NPM1* or *CEBPA* were found to have no significant impact on CPX-351 treatment responses. *FLT3*-ITD-positive AML blasts were also observed to have an increased uptake of CPX-351 as compared with *FLT3*-ITD-negative blasts (**Figure 1**). Overall, the sensitivity of AML blasts to CPX-351 in this *ex vivo* assay was consistent with the observed clinical activity in patient populations (11).

The above findings were corroborated by analysis of AML cell lines (MOLM-13, MOLM-14, and ME1), which also showed increased sensitivity to CPX-351 and increased drug uptake in the presence of *FLT3*-ITD or FLT3-activating mutations, compared with other genetic abnormalities (12). It has been hypothesized that dysregulation of FLT3 signaling may lead to the activation of liposome uptake pathways, resulting in increased sensitivity to CPX-351. Notably, pretreatment of AML cell lines with the FLT3 inhibitor, quizartinib, for 16 h resulted in ~50% of the total cell population exhibiting a decrease in daunorubicin fluorescence, an indicator of drug uptake, suggesting that prolonged FLT3 inhibition may decrease CPX-351 uptake. It follows that alterations in the timing of FLT3 inhibitor treatment may impact effectiveness. Consistent with this, it was found that combining CPX-351 with FLT3 inhibitors (quizartinib and midostaurin) had a synergistic effect when the drugs were used simultaneously or when CPX-351 was scheduled 24 h before the FLT3 inhibitor. However, when FLT3 inhibitors were administered 24 h prior to CPX-351, less synergy was seen, and at certain doses, the effects were antagonistic (12). The authors concluded that these results provide additional evidence that FLT3 activation leads to increased uptake of CPX-351.

## Evidence supporting a role for CPX-351 monotherapy in patients with *FLT3*-mutated AML

In the pivotal phase III study, 22 (16%) patients in the CPX-351 arm and 21 (15%) patients in the 7 + 3 control arm had mutations in the *FLT3* gene (2, 3). Response rates were higher with CPX-351 vs. 7 + 3 in patients with *FLT3* mutations (complete remission (CR)/CR with incomplete neutrophil or platelet count recovery (CRi) rate = 68.2% vs. 23.8%; odds ratio = 6.86; 95% CI = 1.78–26.36; CR rate = 54.5% vs 19.0%; odds ratio = 5.10; 95% CI = 1.29–20.17) (2). Furthermore, *post-hoc* subgroup analysis showed a trend toward improved OS with CPX-351 vs. 7 + 3 in patients with *FLT3* mutations (median OS = 10.25 months vs. 4.60 months; HR = 0.76; 95% CI = 0.34–1.66), although caution should be exerted when interpreting these data due to the small number of patients with baseline *FLT3* mutations included in this analysis. In patients with wild-type *FLT3*, the median OS was 9.33 months with CPX-351 and 5.98 months with 7 + 3 (HR = 0.64; 95% CI = 0.47–0.87). These findings have been supported by data generated in a real-world setting, as outlined below and summarized in **Table 1**.

**Table 1:** Summary of published clinical study data and real-world evidence for use of CPX-351 monotherapy in patients with *FLT3-*ITD/TKD mutations.

Study	Patient population	Patients with *FLT3* mutation (*n*)	CR/CRi rate	OS
Phase III clinical study (CPX-351 vs. 7 + 3)				
Open-label, phase III trial (2, 3)	Newly dx high-risk/sAML (aged 60–75 years) (*n* = 309)	22 (CPX-351); 21 (7 + 3)	68.2% with CPX-351 vs. 23.8% with 7 + 3 (OR = 6.86)	Median 10.25 months with CPX-351 vs. 4.60 months with 7.3 treatment (HR = 0.76; 95% CI = 0.34–1.66)^a^
Real-world studies				
German retrospective study (25 centers) (13)	Newly dx tAML/AML-MRC (*n* = 188)	13 (*FLT3*-ITD)	58% in *FLT3*-ITD mutated vs. 48% of *FLT3*-ITD WT patients (*p* = 0.45)	HR = 0.4 (95% CI = 0.2–1.1; *p* = 0.21)
French retrospective study (12 centers) (14)	Newly dx, untreated t-AML or AML-MRC (*n* = 103)	9 (*FLT3*-ITD); 6 (*FLT3*-TKD)	67% in *FLT3*-ITD mutated vs. 60% in *FLT3*-ITD WT patients (*p* = 0.72); 50% in *FLT3*-TKD mutated vs. 60% in *FLT3*-TKD WT patients (*p* = 0.62)	–
Canadian retrospective study (6 centers) (15)	High-risk patients with t-AML or AML-MRC (*n* = 50)	8 (*FLT3*-ITD); 2 (*FLT3*-TKD); *n* = 5 treated with CPX-351 + midostaurin	–	No difference in OS when stratified by *FLT3*-ITD status (*p* = 0.29)
Italian compassionate use program for CPX-351 (16)	Elderly patients with sAML or t-AML (*n* = 71)	5 (*FLT3*-ITD)	60% in *FLT3*-ITD-positive vs. 70.3% of *FLT3*-negative patients	12-month OS rate: 60% in *FLT3*-ITD-positive vs. 68.3% in *FLT3*-negative patients (*p* = 0.570)

a Post-hoc subgroup analysis.

AML, acute myeloid leukemia; AML-MRC, acute myeloid leukemia with myelodysplasia-related changes; CR, complete remission; CRi, complete remission with incomplete neutrophil or platelet count recovery; dx, diagnosed; *FLT3*, FMS-like tyrosine kinase 3; HR, hazard ratio; ITD, internal tandem duplication; NR, not reached; OS, overall survival; sAML, secondary acute myeloid leukemia; t-AML, therapy-related acute myeloid leukemia; TKD, tyrosine kinase domain; WT, wild type.

A German real-world study of first-line CPX-351 efficacy and safety in adults with newly diagnosed AML-MRC or t-AML (*n* = 188) included 13 patients with *FLT3*-ITD mutations (13). The CR/CRi rate was 58% in patients with *FLT3*-ITD mutations and 48% in patients with wild-type *FLT3*-ITD (*p* = 0.45 for difference). The presence of a *FLT3*-ITD mutation was not found to be a factor impacting OS in univariate analysis (HR = 0.4; 95% CI = 0.2–1.1; *p* = 0.21). In a retrospective study that assessed CPX-351 efficacy and safety in adults with newly diagnosed, untreated t-AML or AML-MRC in France (*n* = 103), the CR/CRi rate for adults with *FLT3*-ITD mutations was 67% (*n* = 6). In patients without *FLT3*-ITD mutations, the CR/CRi rate was 60% (*n* = 53; there was no statistical difference between the groups; *p* = 0.72) (14). In patients with *FLT3*-TKD mutations, the CR/CRi rate was 50% (*n* = 3), and in patients with nonmutated *FLT3*-TKD, the CR/CRi rate was 60% (*n* = 56; *p* = 0.62 for difference) (14). Another real-world study that supports the data from the phase III study described above is a Canadian multicenter study of 50 patients who received CPX-351 for t-AML and AML-MRC, 10 of whom had mutations in *FLT3* (eight ITD and two TKD). Analysis of OS by risk factors showed no differences, including when stratified by the presence of a *FLT3*-ITD mutation (*p* = 0.29) (15). Furthermore, in an Italian compassionate use program that recruited patients aged 52–79 years with secondary AML, three of five patients with a *FLT3*-ITD mutation achieved CR/CRi with CPX-351; the 12-month OS was 60% (16). It is important to note that all real-world studies described here had limited numbers of patients with *FLT3* mutations; however, these findings support a role for CPX-351 as early-line treatment in patients with *FLT3*-mutated AML.

## Combination treatment with CPX-351 + a FLT3 inhibitor in patients with *FLT3*-mutated AML

The phase Ib V-FAST master study (NCT04075747) was initiated to investigate the recommended phase II dose and the safety and tolerability of CPX-351 when administered in combination with targeted agents in patients with AML who are fit to receive intensive chemotherapy (17, 18). Patients with mutated *FLT3* were assigned to receive CPX-351 in combination with the FLT3-inhibitor midostaurin.

Preliminary results from the ongoing V-FAST study suggest that the combination of CPX-351 + midostaurin is a feasible treatment strategy for adults with newly diagnosed AML who have an *FLT3* mutation (17, 18). In total, 23 patients with *FLT3*-mutated AML received CPX-351 + midostaurin treatment. Of these, 18 (78%) had *FLT3*-ITD, and six (26%) had *FLT3*-TKD mutations (one patient had both mutations and was included in both subgroups). Most patients had *de novo* disease (78% of patients with *FLT3*-ITD and 100% of patients with *FLT3*-TKD mutations). The majority of patients achieved CR with CPX-351 + midostaurin, including 14/17 (82.4%) patients with *FLT3*-ITD mutations, and five of six (83.3%) patients with *FLT3*-TKD mutations. Among patients with a CR and known measurable residual disease status (as assessed by multicolor flow cytometry at local laboratories), rates of measurable residual disease negativity after induction were 50% for those with *FLT3*-ITD mutations and 33.3% for those with *FLT3*-TKD mutations. At the time of analysis, 10 of 18 patients (55.6%) with *FLT3*-ITD mutations and two of six patients (33.3%) with *FLT3*-TKD mutation had proceeded to hematopoietic cell transplantation following treatment with CPX-351 + midostaurin. Hematologic recovery times in the V-FAST study were consistent with CPX-351 monotherapy. The overall safety profile was manageable, with serious adverse events reported in 33.3% of patients and no deaths occurring prior to study day 60 (17, 18). These findings are supported by case reports from three patients who received CPX-351 + midostaurin for the treatment of *FLT3* mutation-positive secondary AML. These were a 75-year-old man with AML-MRC and *FLT3-ITD* mutation, a 73-year-old woman with t-AML and *FLT3*-TKD mutation, and a 60-year-old woman with AML-MRC and *FLT3*-TKD mutation. In each case, combination treatment did not cause any unexpected adverse events and resulted in a CR. Overall, the median time to neutrophil recovery (≥ 500/µL) was 31 days, while the median time to platelet recovery (≥ 50,000/µL) was 32 days. OS in the three treated patients was 17, 4, and 12 months, respectively (19). One of the three patients went on to receive an allogeneic stem-cell transplant and remained in CR post-transplant.

## Discussion

This mini-review summarizes the growing evidence supporting a role for CPX-351 in the treatment of AML associated with mutations in *FLT3*. Furthermore, preliminary data from clinical trials and patient case reports suggest that the combination of CPX-351 + midostaurin may represent an additional treatment option for patients with *FLT3* mutation-positive AML. Combination therapies using an induction chemotherapy backbone with targeted agents are increasingly being used in eligible patient populations for the treatment of AML (20). The benefit of combining standard chemotherapy with midostaurin in patients with AML and *FLT3* mutations has been demonstrated previously. In the randomized phase III RATIFY study (NCT00651261), the addition of midostaurin to chemotherapy significantly improved OS and event-free survival in adults with newly diagnosed AML and an *FLT3* mutation compared with placebo (21). The combination of CPX-351 + FLT3 inhibitors for AML will be further investigated in the phase I/II NCT04128748 study (CPX-351 + quizartinib), the phase I/II NCT04982354 study (CPX-351 + midostaurin), and the phase III NCT04293562 study (CPX-351 + gilteritinib), which are currently recruiting patients. Interestingly, preclinical data have suggested a synergistic effect with combination treatment when FLT3 inhibitors were administered after CPX-351 (12). While the use of CPX-351 as a combination therapy with FLT3 inhibitors continues to be evaluated in clinical trials, the pivotal CPX-351 clinical trial and real-world studies have demonstrated that CPX-351 monotherapy may be of benefit in AML patients with FLT3 mutations; no significant differences in CPX-351 treatment outcomes were observed between patients with versus without an FLT3 mutation.

A caveat to the findings described in this review is the relatively small number of patients included in each analysis; further validation of these data is warranted in larger studies. In addition, the data described in this article also do not consider the allelic ratio of *FLT3*-ITD mutations, which is known to have an impact on disease prognosis (7). It remains to be determined whether *FLT3*-ITD^high^ or *FLT3-*ITD^low^ genotypes show differential responses to CPX-351 treatment. However, using allelic ratio testing in clinical practice presents challenges as it is often unavailable to treating physicians, and there is a lack of validated and standardized methods between laboratories. Furthermore, *FLT3-ITD* allelic ratios are no longer part of the European LeukemiaNet risk classification at initial diagnosis for AML (22).

Also worthy of consideration and future study are the mechanisms of resistance to CPX-351 treatment, such as *TP53* mutation (23), and how these may specifically impact patients with *FLT3* mutations.

In summary, the findings reviewed in this article highlight the potential for CPX-351 use in the treatment of patients with AML and mutations in *FLT3*. Combination approaches with CPX-351 and FLT3 inhibitors are supported by preclinical data and initial findings from clinical studies.

## Author contributions

CA: Writing – original draft, Writing – review & editing. VP: Writing – original draft, Writing – review & editing. CR: Writing – original draft, Writing – review & editing.
